# Heart regeneration is regulates by key micro RNAs from fish to mammals: what it can learned about the epicardial cells activation during the regeneration in zebrafish

**DOI:** 10.1038/s41419-018-0609-7

**Published:** 2018-05-29

**Authors:** Nicla Romano, Marcello Ceci

**Affiliations:** Department of Ecological and Biological Sciences, University of Tuscia, Viterbo, Italy

Zebrafish could be an interesting translational model to understand and improve the post-infarction trial and possible regeneration in mammals. The regenerative capacity in mammalian heart is maintained partially in neonatal life^1^ because are still switch-on some phylogenetic-conserved genes; while in adult, it seems to confined in replacing by a few of cardiomyocytes (CM) and by a large amount of fibroblasts^2,3^. In contrast, natural cardiac regeneration appears to be excellent in fish after an injury. In zebrafish, the cardiac environment created by cardiomyocytes, fibroblasts, and non-muscle cells after injury is believed to be critical in facilitating the regenerative response^4^. The epicardium-derived cells and the consequent epicardial cells (EPCs) are essential regulators since they respond to FGFs in both embryogenesis and regeneration processes. EPCs undergo to number of cellular modifications^5,6^ that is required to silenced by miRs that controlled the differentiation of the cells. This downregulation is a necessary event to activate the transition from epithelial to mesenchymal cells, such as cytoskeletal re-arrange and expression of hyaluronan-mediated motility receptor, necessaries to move in the damage site^7,8^. In the heart several molecules are involved in the protein synthesis regulation^9^ where the miRs have a upstream actions. MiR1 and miR133 (a and b) are involved in the activation of fibroblasts in producing FGFs^10,11^, as well as the hypertrophic response of epithelial and muscular cells after injury, to compensate for the loss-of-contractile tissue in mammals, as well as in zebrafish^9,12–14^.

**Fig. 1:**
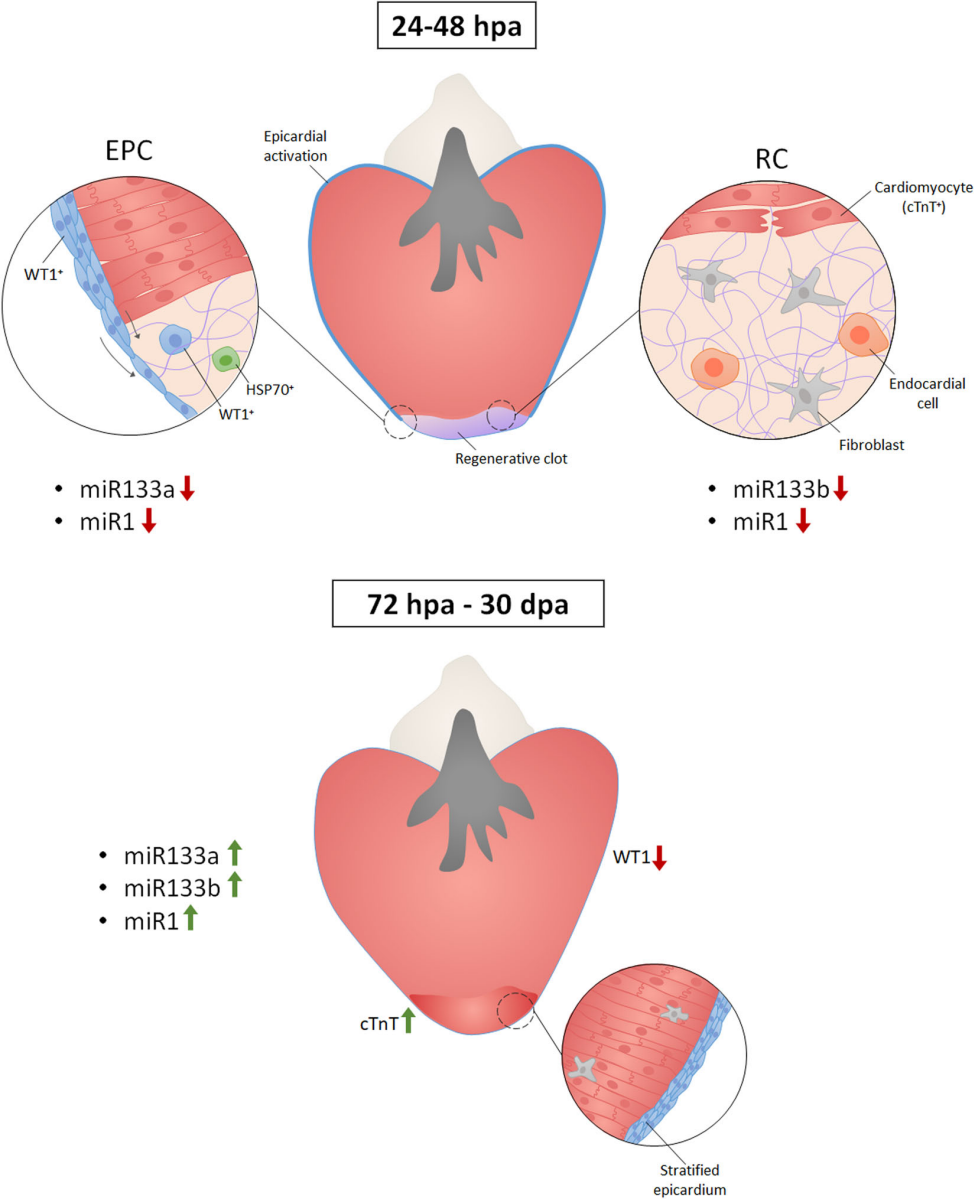
Schematic model that synthesize the miR1 and miR133 actions in blocking the FGF-dependent transduction-pathway in the cells involved in cardiac regeneration: cardiomyocytes (positive to cardio Troponin fraction T, cTNT+), fibroblast, epicardial-derived (positive to Willm’s Tumor 1, WT1+) and endocardial-derived cells. In zebrafish these four types acting in concert for the regeneration by undergoing to a trans-differentiation and/or proliferation. For the first time the research of Ceci et al., has demonstrated the early activation of the heart of zebrafish from 24 h (hrs) post injury due to downregulations of miR1, miR133a, and miR133b. From 72 h onwards, the downregulation of miRs start to invert the trend, because they are expressed and acted on stimulating the tissues to a differentiation. The figure resume the information from ref. ^15^

In the recent research edited in Cell Death and Dicovery^15^, we have explored the time lapse of downregulation of miRs 133a, 133b, and miR1 during the regeneration trial in zebrafish, discovering that the downregulation occur already after 24 h post injury (hpi) and had being become critical during the next 48 hpi (Fig. 1). Moreover, by using laser microdissector method we have demonstrated that this downregulation occur differently in activated EPCs as compare the site of regeneration. Next to the evaluation of miRs expression, we have analysed the expression of embryonal molecules that are re-expressed during the regeneration process: Willm’s Tumor factor 1 (WT1, that is a marker for activated EPCs cells); Heat Shock Protein 70 (HSP70, a chaperon molecule activated during the embryogenesis in a double function:, block the apoptosis and help in the folding/transport of neo-synthesized proteins) and one of the cardiac Troponin complex (cTNT, factor T, is the molecule that adhere to the tropomyosin and is determinant for the correct contraction of the muscle cardiac fibers, it is early expressed and indirectly controlled by miR1) It is probable that the block of myogenic or hyperplastic role of miR1 is crucial in activating the regeneration process. miR133a is probably a key miR that can activate the epicardium because it showed a more significant downregulation already at 1 dpa. Again, the miR133b could be a key miR because of its direct control on the CTGF protein, necessary to regulate the transition in endocardial cells from epithelial to mesenchymal elements. The Fig. 1 give a synthesis of the cited article and details are available in Ceci et al.^15^. This study provides key clues for the experimental early activation of pro-regenerative responses in the heart in zebrafish, and provides crucial insights for the development of therapies targeting heart disease.
